# The effect of osteoporosis and its treatment on fracture healing a systematic review of animal and clinical studies

**DOI:** 10.1016/j.bonr.2021.101117

**Published:** 2021-08-16

**Authors:** E.A. Gorter, C.R. Reinders, P. Krijnen, N.M. Appelman-Dijkstra, I.B. Schipper

**Affiliations:** a Leiden University Medical Center, Departments of Trauma Surgery, P. O. Box 9600, 2300 RC Leiden, the Netherlands; b Internal Medicine, Center for Bone Quality, P. O. Box 9600, 2300 RC Leiden, the Netherlands

**Keywords:** Osteoporosis, Bisphosphonates, Teriparatide, Fracture healing, Non-union, Delayed-union

## Abstract

**Introduction:**

Osteoporosis is characterised by low bone mass and micro-architectural deterioration of bone structure. Its treatment is directed at the processes of bone formation or resorption, that are of utmost importance in fracture healing. We provide a comprehensive review of the literature aiming to summarize and clarify the effects of osteoporosis and its treatment on fracture healing.

**Material and methods:**

A literature search was conducted in PubMed and Embase (OVID version). In vivo animal and human studies on long bone fractures were included. A total of 93 articles were included for this review; 23 studies on the effect of osteoporosis (18 animal and 5 clinical studies) and 70 studies on the effect of osteoporosis treatment (41 animal, 26 clinical studies and 3 meta-analyses) on fracture healing.

**Results:**

In animal fracture models osteoporosis was associated with decreased callus formation and bone growth, bone mineral density, biomechanical strength and delayed cellular and differentiation processes during fracture healing. Two large databases identified osteoporosis as a risk factor for non-union whereas three other studies did not. One of those three studies however found a prolonged healing time in patients with osteoporosis. Anti-osteoporosis medication showed inconsistent effects on fracture healing in both non-osteoporotic and osteoporotic animal models. Only the parathyroid hormone and anti-resorption medication were related to improved fracture healing and delayed remodelling respectively. Clinical studies performed in predominantly hip and distal radius fracture patients showed no effect of bisphosphonates on fracture healing. Parathyroid hormone reduced time to union in several clinical trials performed in mainly hip fracture patients, but this did not result in decreased delayed or non-union rates.

**Conclusion:**

Evidence that substantiates the negative influence of osteoporosis on fracture healing is predominantly from animal studies and to a lesser extent from clinical studies, since convincing clinical evidence lacks. Bisphosphonates and parathyroid hormone may be used during fracture healing, since no clear negative effect has been shown. Parathyroid hormone might even decrease time to fracture union, without decreasing union rate.

## Highlights

• Osteoporosis negatively influences fracture healing in animal models.
• There is no convincing evidence for a similar effect in humans.
• In animals, bisphosphonates delay bone remodelling
• In animals, parathyroid hormone improves fracture healing
• In humans, anti-osteoporotic drugs do not interfere with fracture healing.

## 1 Introduction

Fracture healing is a result of an orchestrated process on cellular and molecular level, and can be divided in direct (primary) and indirect (secondary) fracture healing 1, 2, 3, 4. Direct fracture healing occurs when the fractured parts are anatomically reduced, compressed and rigidly fixated. Indirect fracture healing occurs via four stages in a situation where (micro) movement of the fracture fragments is possible. The four stages are inflammatory response, soft callus formation, hard callus formation and bone remodelling. For both types of fracture healing four elements are essential in order to achieve fracture union: osteogenic cells, the (mechanical) environment, osteoconductive scaffolds and growth factors [4]. Osteoporosis is considered as a possible risk factor for impaired fracture union. Although the mechanical and biological elements involved in fracture healing are affected by osteoporosis, there is still debate whether and to what extent fracture healing might be impaired by osteoporosis 5, 6.

Osteoporosis is a skeletal disorder that is characterised by low bone mass and micro-architectural deterioration of bone structure, resulting in bone fragility and an increased fracture risk [6]. The prevalence of osteoporosis increases with age. Osteoporotic fractures pose an increasing burden on the healthcare system, since the annual number of osteoporotic fractures will rise to 4.5 million in 2025 in the European Union [7] and is estimated to be around 18 million globally in 2040 [8]. In addition, osteoporotic fractures are associated with high rates of morbidity and mortality [8]. Osteoporosis reduces bone strength because cortical bone becomes porous and cortices become thinner, especially in the metaphyseal or metadiaphyseal regions. Unstable and comminuted fracture patterns, short epiphyseal fragments that complicate fracture fixation, impaired healing due to either too unstable or too rigid fixation, decreased holding power of screws in the osteoporotic bone and early implant-bone construct fatigue are biomechanical problems that may lead to implant loosening and loss of fixation in osteoporotic fractures [8]. These potential problems in fracture management add to the effect of osteoporosis on mechanical and biological elements involved in the healing process as described above.

Anti-osteoporotic drugs, especially antiresorptive therapy, are the cornerstone of treatment for osteoporosis. Their anti-resorptive effect has been posed to negatively influence fracture healing while anabolic therapies like teriparatide have been used in studies trying to enhance fracture healing. As literature provides conflicting evidence, we aimed to perform a systematic review of the current literature to elucidate the role of osteoporosis and osteoporosis treatment as potential risk factors for impaired fracture healing in long bone fractures in animal and clinical studies.

## 2 Material and methods

The following search strategy was used in Pubmed: “("Osteoporosis"[Mesh] OR "Osteoporosis, Postmenopausal"[Mesh] OR osteoporosis [tiab]) AND ("Fracture Healing"[Mesh] OR fracture healing [tiab])”, and in Embase (OVID version) “fracture healing.mp AND Osteoporosis.ab,ti.”. The search was conducted at the first of November 2020 and the results were limited to English language articles. Duplicates were removed before applying selection criteria. Two investigators (EAG and CRR) independently assessed the identified titles and abstracts for relevance.

Only in vivo animal, human studies and meta-analyses on long bone fractures were considered for inclusion. In case of animal studies only articles describing an effect of osteoporosis and/or anti-osteoporotic medication on the histological, biomechanical, radiological and/or clinical process of fracture healing were included. Only clinical studies that reported on one or more of the following outcome parameters were included: (radiographic) time to union, incidence of delayed/non-union or union rate. In case of multiple meta-analyses on the same subject, the most recent meta-analysis was included. The full-text articles of potentially eligible studies were obtained and screened using the same inclusion criteria. Reference lists of eligible studies, reviews and meta-analyses were hand-searched to identify further relevant studies meeting the inclusion criteria. The data extraction was performed by one reviewer (EAG).

**Fig. 1:**
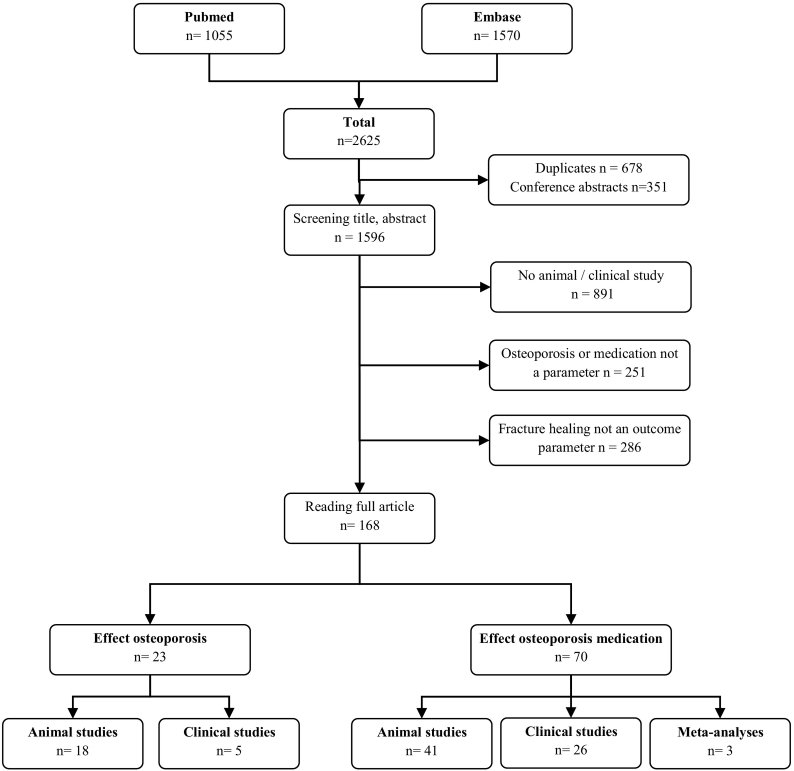
Number and type of studies resulting from the literature search.

Regarding the effect of medication on fracture healing, the results/studies were subdivided based on the mechanism of action (antiresorptive, anabolic or dual), medication group and whether the medication was supplemented in a non osteoporotic or osteoporotic animal model. The article selection process is presented in Fig. 1. The search resulted in a total of 2625 articles, 1055 PubMed and 1570 Embase. After removal of 678 duplicates and 351 conference abstracts, the title and abstract of 1596 articles were screened. Of 168 articles the full text was read. A total of 93 articles were included for this review.

## 3 Results

The results of the 93 included publications are summarized in the Table 1, Table 2, Table 3, Table 4.

**Table 1:** Summary of the effect of osteoporosis on fracture healing in animal studies.

**Osteoporosis**	↓ callus/bone formation 11, 17, 23, 24, 26 ↓ bone mineral content 12, 13, 14, 15, 16, 26 or density 12, 13, 14, 15, 16, 17 ↓biomechanical strength 9, 10, 11, 12, 13, 16, 18, 19, 20, 21, 26 Delay cellular differentiation/processes 9, 12, 13, 15, 16, 18, 21, 24 Radiological delay 12, 16, 18 or no difference 15, 19, 25

↑ significant increased, ↓ significant decreased.

**Table 2:** Summary of the effect of osteoporosis on fracture healing in clinical studies.

Author	Study design	Fracture location	n (patients/control)	Effect	Bias
v Wunnik [29]	Prospective matched controlled	Various	120 (40/80)	No effect on incidence of non-union	Variety of fractures location Small number of patients
Zura [27]	Prospective cohort	Various	56,492 (1440/55,052)	↑ risk of non-union (multivariate analysis OR 1.423, Robust SE 0.108; *p* < 0.001)	Variety of fractures location Insurance database
Zura [28]	Inception cohort	Various	309,330 (15,249/294,081)	↑ risk of non-union (multivariate analysis OR 1.24, 95% CI (1.14–1.34))	Variety of fractures location Insurance database
Nikolaou [30]	Retrospective	Femoral shaft	66 (29/37)	↑ time to union (19.4 weeks vs 16.2 weeks, *p* = 0.02) and delayed union (10/29 vs 4/37 *p* = 0.03) No effect on incidence of non-union	Selection/inclusion Small number of patients No correction for age
Gorter [31]	Retrospective	Subcapital humerus and distal radius	455 (133/322)	No clear effect on delayed or non-union	Retrospective design Small number of patient in subgroup Outcome parameter

↑ significant increased, ↓ significant decreased.

**Table 3:** Summary of effect of osteoporosis treatment on fracture healing in animal studies.

Mechanism	Medication group	Non osteoporotic animal models		Osteoporotic animal models (female)
Antiresorptive	Bisphosphonates	Male ↑ callus formation 32, 33, 38 ↑ biomechanical strength [35] Histologically advanced healing [34] No effect on biomechanical strength 36, 38 No effect on radiological healing [34] No effect on incidence non-union 33, 36 Delay remodelling 33, 35, 37	Female ↑ bone mineral content/density [38] ↑ callus formation [39]	↑ bone mineral content [25] ↑ callus formation 19, 40, 42 ↑ biomechanical strength 19, 40, 41 Histologically advanced healing [40] No effect on biomechanical strength [43] No effect on radiological healing [41] No effect on callus formation [43] No effect on incidence non-union [41] Delay remodelling 25, 41, 42
	Selective estrogen receptor modulator (SERM)	Male –	Female ↑ bone mineral content [38] ↑ biomechanical strength [38] ↑ newly formed bone [44]	↑ callus formation [46] ↑ biomechanical strength [45] Histological advanced healing [45] No effect on callus formation [25] No effect on biomechanical properties [25] Delay remodelling [25]
	RANK ligand inhibitor	Male ↑ biomechanical strength [35] Delay remodelling [35]	Female No effect on callus formation [47] No effect on biomechanical strength [47] Delay remodelling [47]	–
Anabolic	Parathyroid hormone	Male ↑ bone mineral content 50, 54/density 49, 54 ↑ callus formation 49, 50, 51, 52 ↑ newly formed bone 32, 33, 39, 50, 53 ↑ biomechanical strength 49, 50, 51, 53 Histological advanced healing [55] Improved radiological healing [49] Improved union rate [55] No effect on union rate [51]	Female ↑ bone mineral content 38, 56/density [56] ↑ newly formed bone 38, 57 ↑ biomechanical strength 38, 56 Improved union rate [57]	↑ bone mineral content 58, 61/density [61] ↑ callus formation [59] ↑ newly formed bone 61, 63, 64, 65, 66 ↑ biomechanical strength 59, 60 Improved union rate [57] No effect on callus formation [58] No effect on radiological healing [58]
Dual effect	Strontium ranelate	Male No effect on fracture healing [68] No effect on radiological healing [68]	Female ↑ callus formation [38] ↑ bone mineral density [38] ↑ biomechanical strength [38] No effect on biomechanical strength [67]	↑ callus formation 22, 69, 70, 71 ↑ newly formed bone [69] ↑ bone mineral density 69, 70 ↑ biomechanical strength 69, 71 No effect on fracture healing [14]

↑ significant increased, ↓ significant decreased.

**Table 4:** Summary of effect of osteoporosis treatment on fracture healing in clinical studies.

Mechanism	Medication Group	Author	Study design	BMD	Fracture location	n (patients/control)	Drug initiation	Effect
Antiresorptive	Bisphosphonates	Li [72]	Meta-analysis	Mixed		2888		No effect on time to union or union rate
		Colon-Emeric C [74]	RCT	Mixed and unknown	Hip	2127 (1065/1062)	< 90 days vs placebo	No effect on incidence of delayed union or union rate
		Uchiyama [76]	RCT	Mixed	Distal radius	80 (40/40)	<4 d vs >4mo after surgery	No effect on time to union or union rate
		Kim [73]	RCT	Osteoporotic	Hip	90 (30/30/30)	<1wk vs > 1mo vs > 3mo	No effect on incidence of delayed union or union rate
		Gong [78]	RCT	Osteoporotic	Distal radius	50 (24/26)	2wk vs 3mo after surgery	No effect on time to union or union rate
		Vd Poest clement [75]	RCT	Osteoporotic	Distal radius	37 (18/19)	After 2-4wk vs placebo	No effect on time to union
		Duckworth [77]	RCT	Unknown	Distal radius	421 (215/206)	<2wks vs placebo	No effect on union rate
		Shoji [83]	Prospective controlled cohort	Mixed	Distal radius	33 (11/22)	Current vs no use	No effect on union rate
		Hayer [79]	Prospective cohort	Osteoporotic	Hip	43	< 1wk	No effect on time to union
		Koshy [86]	Prospective controlled cohort	Unknown	Distal radius	66 (33/33)	Current vs no use	No effect on time to union
		Rozental [85]	Retrospective	Mixed	Distal radius	196 (43/153)	Current vs no use	Significant increased healing time (55 days vs 49 days)
		Solomon [84]	Retrospective	Mixed	Humerus	891 (81/810)	After fracture vs no use	Significant increased risk on non union (OR2.37, 95% CI 1.13–4.96)
		Lim [80]	Retrospective	Osteoporotic	Hip	130 (29/101)	Current or previously vs no use	Significant increased risk of delayed union after 3 months (union rate after 3 months 21/29 Vs 91/101), no difference after 1 year
		Seo [82]	Retrospective	Osteoporotic	Proximal humerus	82 (34/48)	<2wks vs >3mo after surgery	No effect on time to union or union rate
		Cho [81]	Retrospective	Osteoporotic	Hip	284 (102/89/93)	1wk vs 1 month vs 3 months	No effect on time to union
	RANK ligand inhibitor	Adami [87]	RCT	Osteoporotic	Nonvertebral fractures	7808 (3902/3906)	Denosumab vs placebo	No effect on delayed and non union
Anabolic	Parathyroid hormone	Han [88]	Meta-analysis	Osteoporotic and unknown	Hip	607		Significant reduced time to union (OR−1.95; 95% CI: −3.23–−0.68), no effect on union rate after 3 or 6 months.
		Hong [95]	Meta-analysis	Mixed and unknown		524		(−3.05, 95% CI −5.96 to −0.14) reduced time to union, no effect on union rate
		Peichl [96]	RCT	Osteoporotic	Pubic	65 (21/44)	100 μg PTH 1–84 vs control	Significant reduced time to union (7.8 weeks vs 12.6 weeks)
		Kanakaris [97]	RCT	Osteoporotic	Hip		vitDcalcium Vs vitD calcium & bisphosphonates Vs vitD calcium & tereparatide	Prematurely ended due to slow patient accrual
		Bhandari [93]	RCT	Unknown	Hip	159 (78/91)	20 μg teripratide vs placebo	Prematurely ended due to slow patient accrual, but no difference radiological union rate
		Chesser [94]	RCT	Unknown	Hip	29 (15/14)	Teriparatide vs control	No difference in union rate
		Aspenberg [99]	RCT	Unknown	Distal radius	102 (34/34/34)	20 μg vs 40 μg teripartide vs placebo during 9 weeks	20 μg significant reduced time to union (7.4 weeks vs 9.1 weeks), however 40 μg did not
		Almirol [98]	RCT	Unknown	Stress fracture leg	13 (6/7)	20 μg teripratide vs placebo	No difference in radiographic fracture healing
		Huang [89]	Retrospective	Osteoporotic	Hip	189 (83/47/59)	vitD calcium Vs vitD calcium and teripartide Vs previous on alendronate after fracture on vitD calcium and teripartide	Significant reduced time to union (12.3 weeks vs 13.6 weeks), no effect on incidence of delayed union
		Huang [90]	Retrospective	Osteoporotic	Hip	73 (29/44)	20 μg teriparatide vs no	Significant reduced time to union (11.2 weeks vs 14.3 weeks), no effect on incidence of delayed- or non- union
		Kim [91]	Retrospective	Osteoporotic	Hip	112 (52/60)	20 μg teriparide vs nothing	Significant reduced time to union (12.1 weeks vs 14.8 weeks), no effect on incidence of non union
		Kim [92]	Retrospective	Osteoporotic	Hip	96 (50/46)	Daily teriparide vs nothing	No difference in time to union
Dual effect	Strontium ranelate	Scaglione [101]	RCT	Unknown	Distal radius	40 (20/20)	vitD calcium Vs vitD calcium and strontium ranelate	No effect on radiological follow-up

BMD Bone mineral density; RCT Randomized controlled trial.

### 3.1 Effect of osteoporosis on fracture healing - animal studies (Table 1)

A total of 18 prospective animal studies were found describing the effect of osteoporosis on fracture healing. Overall, in animal studies osteoporosis was found to negatively influence fracture healing in the majority of studies. Delayed cellular processes, decreased callus formation and mineralization may be the possible explanation of the observed decrease of biomechanical strength. No clear effect of osteoporosis was found in radiological follow-up.

In mice, the effect of osteoporosis on the fracture healing of the femur was investigated, the micro-CT analysis showed impaired healing in the osteoporotic group [9]. In another genetic osteoporotic mice model with a femoral fracture age dependent differences were found: bending stiffness, callus size, and callus tissue distribution were not altered in 5-month-old osteoporotic mice compared to non-osteoporotic mice. In 10-month-old osteoporotic mice however bending stiffness was significantly reduced and callus size was increased compared to non-osteoporotic mice, indicating delayed fracture healing, possibly explained by an increased osteoclast activity in the 10-month-old [10].

In rats, several studies showed that in the osteoporotic group the total callus, there was less bony callus and newly formed bone [11] as well as the bone mineral content and bone mineral density was reduced at the fracture site 12, 13, 14, 15, 16. Other studies showed that the presence of osteoporosis had a negative impact on the quality and quantity of callus during early fracture healing 12, 17 and biomechanical testing 11, 12, 13, 16, 18, 19, 20, 21, 22. Another study performed in rats with a tibial bone defect showed that osteoporosis resulted in significantly less newly formed bone, a higher amount of granulation tissue and immature newly formed bone, compared to rats without osteoporosis [23]. Histological evaluation revealed a delay in the cellular differentiation processes of chondrocytes during fracture healing 12, 13, 18. In ovariectomized rats with a femoral osteotomy, histological analyses showed less mature consolidation [21], significantly reduced bone volume was found at the gap [24], the gap contained more osteoclasts [24] and the gap was filled with scattered smaller bone trabeculae [24] compared to non-ovariectomized rats. But the microcomputer tomography (μCT) showed no difference in consolidation [21]. However Gauo et al. [19] found no significant differences in bone microarchitecture on the micro CT between the osteoporotic and non-osteoporotic rats 12 weeks after fracture induction. Coa et al. [25] also did not find impaired callus formation or biomechanical strength.

Even in a larger animal model similar results were found. In fourteen sheep with a tibial shaft osteotomy osteoporosis resulted in impaired fracture healing with respect to callus formation, mineralization, and biomechanical properties [26].

Two studies showed that fracture healing in osteoporotic animals was also radiographically lagging behind 12, 18, or described clear differences in union rate (59% osteoporotic group vs 89% in the control group) after 8 weeks [16]. One study found that the fracture was partly united compared to a clearly present fracture gap in osteoporotic animals at 4 weeks. However, after 12 weeks bone union was observed in both groups [19]. Kubo et al. [15] also showed no radiological differences in femoral fracture healing between ovariectomized and non-ovariectomized rats. Another study in ovariectomized and non-ovariectomized rats did not show a clear impairment of radiological healing [25].

### 3.2 Effect of osteoporosis on fracture healing - clinical studies (Table 2)

No meta-analyses were found investigating the effect of osteoporosis on fracture healing. Five clinical studies, 3 prospective and 2 retrospective, were found investigating the effect of osteoporosis on fracture healing. Overall, two large databases identified osteoporosis as a risk factor for non-union while three other studies did not. One of those three studies however found a prolonged healing time in patients with osteoporosis.

In two large database studies osteoporosis was identified as a risk factor for non-union 27, 28. In one analysis of a national insurance database, 47,437 patients were included in 12 months with 56,492 fractures for which a non-union was registered in 2.5%. Sixty potential patient characteristics and co morbidities for non-union were assessed and osteoporosis was identified as a risk factor for non-union [27]. In an even larger database using patient-level health claims, 309,330 fractures in 18 bones with 15,249 non-unions (4.9%) were registered in 12 months. Again osteoporosis was identified as an influencing factor [28]. In a matched case-control study, on prospective gathered data, of 40 patients with fracture non-union and 80 patients without a fracture non-union a regression analysis was performed to investigate whether the presence of osteoporosis attributed to the non-union, but did not detect any correlation [29].

In a small study, 29 patients, aged >65 years, with a femoral shaft fracture and radiological evidence of osteoporosis based on the Singh index were retrospectively compared with 37 subjects, aged between 18 and 40 years, without radiological evidence of osteoporosis. A prolonged union time (19.38 ± −5.9 weeks vs 16.19 ± −5.07 weeks, *p* = 0.02) with more delayed unions (>24 weeks) was described (10/29 vs 4/37 *p* = 0.03) in the older group with osteoporosis. However, all fractures healed within 32 weeks [30]. Although patients with known metabolic disorders were excluded, no analysis to unknown metabolic disorders was performed nor correction was performed for age. A retrospective study on subcapital humerus fractures (*n* = 311) and distal radius fractures (*n* = 150) found a seemingly negative association, but no statistically significant evidence that osteoporosis was associated with delayed or non-union [31].

### 3.3 Effect of anti-osteoporosis medication on fracture healing - animal studies (Table 3)

A total of 41 studies were found describing the effect of anti-osteoporosis medication on fracture healing in both osteoporotic and non-osteoporotic animal models. The studies were subdivided based on the working mechanism of the drug (antiresorptive or anabolic), type of medication and whether the medication was studied in a non osteoporotic or osteoporotic animal model. Both male and female species were used for non-osteoporotic models, whereas only female species were used in the osteoporotic animal models. Overall, inconsistent effects on fracture healing in both non-osteoporotic and osteoporotic animal models were observed. Antiresorptive drugs, bisphosphonates in particular, resulted in delayed remodelling of callus in both models. Parathyroid hormone was related to improved fracture healing.

#### 3.3.1 Antiresorptive medication

##### 3.3.1.1 Bisphosphonates

In male non osteoporotic rats models increased callus volume [32], hard callus bone mineral content [33], histologically more advanced healing [34] and increased mechanical strength 33, 35 were found after supplementation of bisphosphonates. Another study found no effect on mean elastic modulus and hardness of the callus tissue in male rats [36]. In male rat models delayed fracture healing [37] and remodelling 33, 35, 37 was found after supplementation of bisphosphonates, but also no effect on union rate was described. 33, 36 Aydogan et al. [34] found no effect of on fracture healing in rats with a femur fracture in radiological follow-up. In female non osteoporotic rat models with a femoral fracture, treatment with bisphosphonates increased bone mineral content [38], bone mineral density [38] and callus volume [38] compared to wild type rats [38] and local application of bisphosphonates resulted in more callus formation [39].

Osteoporotic models – In rats with a tibial fracture, administration of zoledronic acid resulted in increased biomechanical strength, more callus as well as thicker and more mature bone trabeculae, and in both the zoledronic acid group and the control group there was complete healing [40]. Bisphosphonates in rats with a femoral fracture increased the mechanical strength of the callus [41] and hard callus bone mineral content [25]. Mice with a femoral osteotomy treated with alendronate showed an increase in newly formed bone at the defect site [42]. Local application of bisphosphonates at the fracture site in rats improved bone microarchitecture, mechanical character and resulted in more callus [19]. However, one study found that the administration of alendronate in osteoporotic rats with a metaphyseal tibial fracture did not influence the process of fracture healing quantitatively or qualitatively [43]. Despite the observed positive effects of bisphosphonates other studies in rats found suppressed callus remodelling [41], delayed remodelling [25] and suggested that continuous administration might be detrimental to bone repair [42].

##### 3.3.1.2 Selective estrogen receptor modulator (SERM)

In a comparative study of 60 non osteoporotic female mice the administration of raloxifen resulted in enhanced fracture healing and earlier complete bony bridging of the femoral osteotomy gap compared to mice not receiving raloxifen [44]. In non osteoporotic female rats, raloxifen treatment increased bone mineral content, bone mineral density and biomechanical properties significantly, even though no greater bone volume on CT scans compared to other treatment groups was observed. [38]

Osteoporotic models – In rat models the effect of raloxifen on peri-implant bone healing was investigated by Ramalho-Ferreira et al [45]. They showed improved fracture healing compared to osteoporotic rats not receiving raloxifen and similar histological and biomechanical values compared to the non-osteoporotic rats. In rats with a metaphyseal tibial fracture raloxifen in combination with estrogen resulted in improved fracture healing with regard to callus formation [46]. On the other hand, no effect on callus formation or biomechanical properties was found by Cao et al. [25] in female rats and raloxifen was not found to be more inhibitory on the process of fracture healing due to inhibited resorption activity and reduced remodelling.

##### 3.3.1.3 RANK ligand inhibitor

Non osteoporotic animal models – Ulrich-Vinther et al. [47] showed that OPG (natural decoy binding protein of RANKL) treatment did not influence callus formation or mechanical strength in female rats, however during the remodelling phase it impaired the normal remodelling and consolidation process. In a mouse model treatment with RANK-ligand inhibitor resulted in reduced bone resorption during fracture healing without being detrimental to fracture healing [48]. Gerstenfeld et al. [35] found an increased mechanical strength in male mice after treatment with denosumab, but showed delayed callus remodelling.

#### 3.3.2 Anabolic medication

##### 3.3.2.1 Parathyroid hormone

Non osteoporotic male animal models – Treatment with a PTH receptor agonist resulted in increased callus osteogenesis, improved fracture bridging, greater bony callus size and density, improved biomechanical stability and more callus on radiological follow-up in male rats with a femoral fracture [49]. Also in other animal fracture studies PTH supplementation resulted in complete consolidation [38], enhanced biomechanical strength 38, 50, 51, bone mineral content 38, 50, denser callus [52] and more callus 50, 51 or newly formed bone 38, 50. In rats with type 2 diabetes and a femoral fracture the administration of PTH resulted in increased bone formation, mineralization and mechanical strength [53]. In rats with a large sized osteotomy in the femur local and systemic PTH was applied and resulted in higher bone mineral density and bone mineral content at the osteotomy site compared to rats without treatment [54]. With regards to fracture union, in a rat model with an open or closed femoral osteotomy the treatment with PTH did not result in an increased union rate [51]. In a femoral atropic non-union model in mice, treatment with PTH showed higher rates of bony union and reduced mean gap size with cortical bridging with mature bone and relatively little callus on histological analysis [55].

Non osteoporotic female animal models – Also in female animal fracture studies PTH supplementation resulted in complete consolidation [38], enhanced biomechanical strength [56], bone mineral content [56], increased BMD [56] and Nozaka et al [57] found in rats with a proximal tibial osteotomy increased cancellous bone formation and improved union rate.

Osteoporotic animal models – In ovariectomized rats Ellegaard et al. [58] showed that treatment with parathyroid hormone (PTH) resulted in a non-significantly increased amount of callus after 4, 6 weeks and no difference after 8 weeks. Also PTH supplementation resulted in enhanced biomechanical strength 59, 60, bone mineral content 58, 61, increased BMD 58, 61 and more callus 58, 59 or newly formed bone 60, 61, 62. In rats, the administration of parathyroid hormone improved the differentiation and proliferation of hypertrophic chondrocytes [63], and newly formed trabecular bone was increased [63] as well as the cancellous bone formation 57, 63. The finding that PTH enhances bone formation was supported by other studies in which also local beta-tricalcium phosphate was applied at the defect site 64, 65. A combination of teriparatide and anti-RANKL monoclonal antibody in mice resulted in accelerated regeneration of cancellous bone during fracture, however no effect was found on cortical bone regeneration or cortical bone thickness [66]. In rats with a cancellous bone osteotomy of the tibia the administration of parathyroid hormone improved union rate [57].

#### 3.3.3 Antiresorptive and anabolic medication

##### 3.3.3.1 Strontium ranelate

Non osteoporotic animal models – Administration of strontium ranelate in a female fracture animal model resulted in increased bone formation, bone mineral density, higher mechanical strength and improved callus formation [38]. One study found a positive effect on callus volume and bone mineral content after 3 weeks but no effect after 8 weeks and no effect on maximum load or stiffness at the fracture site in female rats [67]. Also in male rats, Cebesoy et al. [68] found no beneficial effects of strontium ranelate on radiological or histopathological fracture healing.

Osteoporotic animal models – Administration of strontium ranelate in several fracture or osteotomy animal models resulted in increased bone formation 22, 69, bone mineral density 22, 69, 70, higher mechanical strength 22, 69, 71 and fracture stiffness [71], improved callus formation 69, 70, 71. However, one study showed that administration of strontium ranelate with insulin compared to only insulin in ovariectomized diabetic rats did not display a significant advantage regarding fracture healing [14].

### 3.4 Effect of anti-osteoporosis medication on fracture healing - clinical studies (Table 4)

A total 26 clinical studies and 3 meta-analyses were found describing the effect of anti-osteoporosis medication on fracture healing. The studies were subdivided based on their effect (antiresorptive or anabolic), medication group and whether the medication was supplemented in a osteoporotic or non-osteoporotic patients. Overall, no clearly positive nor negative effect could be found of antiresorptive medication on fracture healing. With regards to the anabolic medication, recombinant parathyroid hormone decreased time to union in several studies without an effect on delayed or non union rates. One study was found on strontium ranelate, which showed no effect.

#### 3.4.1 Antiresorptive medication

##### 3.4.1.1 Bisphosphonates

A meta-analysis of the effect of bisphosphonates on fracture healing of 10 RCTs including 2888 osteoporotic and non-osteoporotic fracture patients was performed by Li et al. [72]. No effect on fracture healing time nor on delayed or non-union was found [72]. This meta-analysis included all our identified RCTs 73, 74, 75, 76 except for the studies performed by Duckworth et al. [77] and Gong et al. [78]. Their RCTs on the effect of bisphosphonates on the healing of a distal radius fracture also showed no difference in mean time to radiographic union [78] or union rate 77, 78.

Osteoporotic fracture patients – Gong et al. [78] investigated the effect of bisphosphonates on the healing of a distal radius fracture in a RCT and found no difference in mean time to radiographic union or union rate. In a prospective cohort study performed with 43 hip fracture patients a single dose of zoledronic acid did not affect radiological fracture union [79]. However, a retrospective analysis among 130 patients with a hip fracture showed that the preoperative use of bisphosphonate (*n* = 29) related to less fracture union after 3 months compared to no bisphosphonate use (72.4% vs 90.1%), but no differences in union rates were found after one year [80]. Cho et al. [81] retrospectively investigated in 284 hip fracture patients whether administration of bisphosphonates after 1 week, 1 month or 3 months influenced fracture healing time. They found no difference in time to union and no cases of non union. In a retrospective study among 82 patients with a operated proximal humerus fracture early initiation of bisphosphonates (<2 weeks) versus late initiation (> 3 months) was investigated, and no difference in union time (6.3 vs 6.6 weeks) or union rate was found [82]. Overall, only one retrospective study found an increased risk on delayed unions based on a difference in union rates after 3 months, without a difference after one year [80]. On the other hand 3 RCTs, one prospective trial and 2 retrospective trials found no effect (Table 4).

Osteoporotic and non osteoporotic fracture patients – One prospective study included 33 patients with a distal radius fracture and found no effect on union rate or function [83]. In a nested case-control study from a large insurance database, 81 patients who underwent an operation for fracture non-union of a humeral fracture were compared with 810 patients without a humeral fracture non union. A multivariate conditional logistic regression analysis showed that post-fracture bisphosphonate use resulted in an increased risk of non-union (RR = 2.37, 95% CI 1.13–4.96), but pre-fracture use did not (RR = 0.84, 95% CI 0.19–3.74) [84]. In patients without previous fractures or osteoporosis also no effect was found. Although not considered clinically relevant by the authors, one retrospective study on distal radius fractures found an increased healing time (55 days vs 49 days) [85].

Osteoporosis status unknown – The randomized controlled trial by Duckworth et al. [77] found no effect of bisphosphonates on union rate in the healing of a distal radius fracture. Also a retrospective study in patients with a distal radius showed no effect of bisphosphonate on the occurrence of radiological or clinical delayed union [86].

##### 3.4.1.2 RANK ligand inhibitor

In the Freedom trial almost 8000 postmenopausal women >60 years with osteoporosis were randomized to receive 60 mg of denosumab every six months for three years or a placebo. In a sub-analysis of fracture healing among 851 non-vertebral fracture patients (386 in the denosumab group and 465 in the placebo group), delayed union was only reported in two patients (0.5%) in the denosumab group and five patients (1.1%) in the placebo group. No non-unions and one non-union were reported in the denosumab group and placebo group respectively [87].

#### 3.4.2 Anabolic medication

##### 3.4.2.1 Recombinant parathyroid hormone

A recent meta-analysis on the effect of teriparatide on fracture healing in hip fracture patient analysed all 2 RCTs and 4 retrospective studies on hip fracture patients that we identified. [88] Teriparatide was found not to affect union rate, due to study heterogeneity and various sources of biases the limited evidence found did not support the hypothesis that teriparatide improves fracture healing in hip fractures [88]. They included four studies performed in an osteoporotic fracture population 89, 90, 91, 92 and two with an unknown osteoporosis status 93, 94. Another meta-analysis in 2019 investigated the efficacy and safety of r-PTH in fracture healing [95]. This meta-analysis included the eight RCTs that were identified in the present search; three studies were performed in an osteoporotic fracture population 89, 96, 97, in four studies osteoporosis status was unknown 93, 94, 98, 99 and one including both osteoporotic and non osteoporotic patients [100]. Three studies found reduced radiographic time to fracture healing in subjects using teriparatide, although heterogeneity within the studies was high. Four studies found no difference in union rate, again with a high heterogeneity [95]. Remarkable, two or the eight performed RCTs had significant problems with patient recruitment and completion of follow up 93, 97. Of these, only Bhandari et al. [93] analysed their data but were underpowered with 159 patients showing no difference regarding radiographic fracture healing. Among the six remaining RCT's, one was a pilot study among 29 hip fracture patients and found no difference in union rate [94]. Of the remaining five RCTs, only one found a positive effect of recombinant parathyroid hormone. In this randomized study with 65 patients with a pubic bone fracture, daily supplementation of recombined parathyroid hormone 1–84 reduced the mean time to fracture healing compared to no medication (7.8 weeks vs 12.6 weeks, *p* < 0.001). After eight weeks all fractures (*n* = 21) in the treatment group were healed and only 4/44 fractures in the control group were healed (p < 0.001) [96].

Four remaining retrospective studies in hip fracture patients, were also analysed in the meta-analysis of Han et al. [88]. Three out of four found a reduced time to union in the group of patients treated with teriparatide 89, 90, 91, while one study did not find a difference in fracture healing time [92]. Despite the reduced time to union, none of these three studies found a difference in the occurrence of delayed or non-unions 89, 90, 91.

Concerning osteoporotic fracture patients, one RCT and 3 retrospective studies found reduced times to fracture union without an effect on union rate. One retrospective study found no effect.

#### 3.4.3 Antiresorptive and anabolic medication

##### 3.4.3.1 Strontium ranelate

One study evaluated the effect of strontium in fracture healing. In a RCT, 40 nonoperatively treated distal radius fracture patients with an unknown osteoporosis status received either supplementation with vitamin D and calcium or supplementation with vitamin D, calcium and strontium ranelate. No differences in radiological follow-up up, clinical evaluation, and ultrasonography of the callus were found between the two groups [101].

## 4 Discussion

The aim of this systematic review was to elucidate the relationship between osteoporosis and its treatment on fracture healing. In animal studies osteoporosis negatively influenced fracture healing in the majority of studies, with regard to cellular processes, callus formation, mineralization and biomechanical strength. In human studies this evidence was not convincing, although there seemed to be a tendency towards a negative influence of osteoporosis on fracture healing with prolonged healing time and increased risk on non-union. Inconsistent effects of anti-osteoporosis medication on fracture healing in both non-osteoporotic and osteoporotic animal models were observed. Antiresorptive medication, bisphosphonates in particular, resulted in delayed remodelling of callus in both models. Teriparatide was found to enhance fracture healing in animal models. In clinical studies however, no clear negative effect of bisphosphonates were found on time to union and on increased delayed or non union rates. Recombinant parathyroid hormone did seem to decrease time to union without an effect on delayed or non union rates.

The effect of osteoporosis on fracture healing in animal models was primarily investigated in rats and mice. The majority of the results suggested a negative biomechanical or histopathological influence of osteoporosis on fracture healing 9, 10, 11, 12, 13, 14, 15, 16, 17, 18, 19, 21, 23, 24, 26, whereas only three studies found radiological evidence of delayed union 12, 16, 18. This may suggest that local signs of impaired or lagging fracture healing cannot always be radiologically objectivated, which has to be taken into mind while interpret the results. A potential limitation of some studies is the use of animal models with induced osteoporosis. Animals must receive treatments in order to produce a state of low bone mineral density or to become osteoporotic. All studies used ovariectomized animals to create an animal osteoporosis model, except for one study, which used a genetically induced osteoporosis model. [10] In six studies complementary diet was used after ovariectomy 13, 14, 15, 22, 24, 26. Since this is not a natural process in animals, interference with fracture healing could occur. Nevertheless, these models are standardly used for basic research on human biological processes. Additional human factors in fracture healing do not impair these models in such a way that results from animal based studies on osteoporosis have become meaningless. Another point of interest is the lack of a uniform definition of osteoporosis in these animals models. In 11 studies the BMD was checked with a DEXA-scan 11, 13, 15, 16, 18, 21, 22 or micro-CT 9, 24, 25, 26, before the experiments to investigate the effect of osteoporosis on fracture healing were started. One study defined osteoporosis as a BMD ≥2.5 standard deviation (SD) lower than the BMD of the control group [13], whereas another study used a definition in which the BMD should be significantly lower than that of the control group [11]. However, the majority of the studies did not define animal osteoporosis and only described a significant lower BMD in the ovariectomized population 9, 12, 15, 16, 18, 21 compared to the control group by, or diagnosed osteoporosis by the means of a DEXA without providing further details [22]. In case of micro-CT no clear definition of osteoporosis was defined either, but changes of bone architecture were described used to identify osteoporosis; less trabecular bone, disorganized trabecular architecture, expanded marrow cavities and thinning cortical bone [9].

Only five studies investigating the influence of osteoporosis on fracture healing in humans were found. Nikolaou et al. [30] found an increased time to union and delayed union rates, but classified osteoporosis patients based on X-rays (Singh index) and not on Dexa-scan or diagnosed by an endocrinologist. Two large database studies which found a negative effect of osteoporosis 27, 28 might show the power of big data analyses, since the three smaller studies found no clear effect on the incidence of non-union possibly due to lack of statistical power. However, caution is warranted in interpreting these results, as stated by the authors. These large databases were based on claims by patients, often the codes were imprecise, patients were not followed prospectively for a specific outcome and also data was missing. Zura et al. [28] performed the only clinical study that included the use of anti-osteoporosis medication as a variable in their analysis and indeed identified this as a risk factor. The study by Gorter et al. [31] was retrospective, in which only in a small number of patients radiological follow up was available and the possible effect of osteoporosis treatment was not taken into account. More prospective studies like that of Van Wunnik et al. [29] are needed in order to elucidate whether osteoporosis has a negative influence on fracture healing.

In both the non-osteoporotic and osteoporotic animal models, anti-osteoporosis medication was found either to improve or not to influence fracture healing. Also no convincing difference was found between studies performed in male versus female non-osteoporotic animal models. In order to achieve full fracture healing, resorption of the newly formed callus occurs during the remodelling phase. As might be expected some studies on antiresorptive medication, which counteracts resorption, found a negative effect on the remodelling phase of fracture healing 25, 33, 35, 41, 42, 47. There was however no evidence that this negatively influenced the healing process or biomechanical properties of the fracture. Parathyroid hormone showed in both animal models predominantly a positive effect on callus formation, bone mineral content, biomechanical strength and improved union rates in several studies.

Compared to clinical studies on osteoporosis, remarkably more clinical data was available on the effect of anti-osteoporosis medication on fracture healing. Although the studies included typical fragility and osteoporotic fractures, not every study included only patients with a T score < −2.5 and in some studies the information on the BMD was missing at all. None of the studies was performed in non-osteoporotic patients only. Studies on bisphosphonates were predominantly performed in hip and distal radius fracture patients and no effect on fracture healing was found. Especially no clear evidence of delayed union was reported, which might be expected based on the results found in animal models. In case of parathyroid, predominantly hip fracture patients were studied, and the results were more in line with the data found in animal models. Parathyroid hormone seems to improve time to union, however no clear effect on delayed union or non union rates was found. Both meta-analyses showed a high heterogeneity in the included studies due to differences in study design, different BMD groups and fracture locations. Parathyroid hormone supplementation has also been investigated in case of non-union treatment. A recent review concluded that teriparatide could be effective in the treatment of non-unions, when general principles of non-union and infections were dealt with [102]. On the other hand, the positive effects of treatment with teriparatide in order to improve fracture healing in atypical fractures have not been established. 102, 103, 104, 105, 106, 107, 108 Only six RCTs investigated medication versus placebo 74, 75, 77, 87, 93, 98, in other RCTs patients were randomized between early initiation versus late initiation of medication 73, 76, 109 or patients were randomized between receiving the medication or not 96, 97, 100, 101. In order to unambiguously establish the effect of the treatment, a comparison with a placebo should be considered the preferred design. Nevertheless, in the three RCTs comparing early versus late initiation, late initiation was thus late that most of the fracture had already healed. The effect of the medication on fracture healing could be neglected and these patients could be considered as a control group without treatment. The meta-analyses of Han et al. [88] and Li et al. [72] were well performed, whereas the meta-analysis of Hong et al. [95] included also a retrospective study while a randomized study design was an inclusion criterium. Unfortunately for the statistical power, a large number of RCTs and even retrospective studies were performed in a small number of patients despite the fact that an osteoporotic fracture is common. Distal radius fractures have a high union rate and hip fracture patients are often lost to follow-up in prospective studies as shown by Bhandari et al. [93] and Kanakaris et al. [97]. Futures studies should preferably also include large osteoporotic populations of patients with fractures that are known to be associated with a relatively high non-union rate.

Our aim was to provide a systematic review of the current literature in an attempt to elucidate the role of osteoporosis and osteoporosis treatment as potential risk factors for impaired fracture healing in animal and clinical studies. Due to the considerable number of agents that have been studied in different species and patient populations using different study designs, fracture locations and outcome parameters, a meta-analysis was considered not feasible.

In general, one might question the clinical relevance of the shorter radiological union times found in several studies on recombinant parathyroid hormone supplementation 88, 89, 90, 91, 95, 96, 99. Additional data about the clinical and patient-reported outcomes should be provided in order to assess the relevance of this radiological outcome. If a shorter time to radiological union does not influence clinical and patient-reported outcomes, nor does it influence fracture treatment or result in decreased risk on a delayed- or non-union, the clinical relevance of this finding could be deliberated.

In conclusion, animal studies suggest that osteoporosis negatively influences fracture healing. Clinical studies also show a possible negative tendency, but the evidence is still not convincing. In animal models anti-resorptive medication delayed fracture remodelling and teriparatide was related to improved fracture healing, but no clear negative influence of anti-osteoporosis medication on fracture healing could be determined in fracture patients. Recombinant parathyroid hormone did seem to decrease time to union without an effect on delayed or non union rates. Based on this evidence, clinicians should not treat fractures differently in case of osteoporosis and initiate or continue anti-osteoporotic medication in osteoporotic fracture patients without restraint.

## CRediT authorship contribution statement

**Gorter**; Conceptualization, Methodology, Investigation, Data Curation, Writing - Original Draft. **Reinders**; Data Curation, Writing - Review & Editing. **Krijnen**; Conceptualization, Writing - Review & Editing. **Appelman-Dijkstra**: Writing **-** Review & Editing. **Schipper**; Conceptualization, Writing - Review & Editing, Supervision.

## Declaration of competing interest

None.
